# Risk factors for diarrhea hospitalization in Bangladesh, 2000–2008: a case-case study of cholera and shigellosis

**DOI:** 10.1186/1471-2334-14-440

**Published:** 2014-08-15

**Authors:** Danny V Colombara, Abu SG Faruque, Karen D Cowgill, Jonathan D Mayer

**Affiliations:** Department of Epidemiology, University of Washington, Seattle, WA USA; International Centre for Diarrheal Disease Research, Dhaka, Bangladesh; Department of Global Health, University of Washington, Seattle, WA USA; Seattle University College of Nursing, Seattle, WA USA

**Keywords:** Cholera, Shigellosis, Epidemiology, Bangladesh

## Abstract

**Background:**

Cholera and shigellosis are endemic on the Indian subcontinent. Our objective was to identify cholera-specific risk factors distinct from shigellosis risk factors.

**Methods:**

We conducted a case-case study among hospitalized diarrheal patients, comparing those with cholera and shigellosis in International Centre for Diarrhoeal Disease Research, Bangladesh (icddr,b) hospitals in Matlab (rural) and Dhaka (urban) between January 1, 2000 and December 31, 2008.

**Results:**

Multivariable Poisson regression models revealed that having more than nine years of education, compared to no education, was associated with a 39% (adjusted Risk Ratio [aRR] = 0.61, 95% confidence interval [CI]: 0.40-0.93) decreased risk for cholera hospitalization in Matlab and a 16% (aRR = 0.84, 95% CI: 0.75-0.94) decreased risk in Dhaka. Having a family member with diarrhea in the past seven days increased cholera hospitalization risk by 17% (aRR = 1.17, 95% CI: 1.09-1.26) in Matlab.

**Conclusions:**

Further studies are needed to elucidate the pathway through which education impacts cholera risk in order to create targeted interventions in cholera-endemic areas. Interventions seeking to reduce transmission and facilitate hygienic practices among family members of index cases with diarrhea should be considered, especially in rural cholera endemic settings.

## Background

Cholera is a diarrheal disease caused by infection with *Vibrio cholerae* bacteria. Since 1817 the world has endured a series of cholera pandemics [1, 2]. The seventh pandemic, which started in the early 1960s, appeared to be waning at the turn of the millennium, but has since developed new vigor. The global case fatality rate, which was 1.3% in 2011 [3], has remained relatively constant over the last 10 years, but absolute cholera-associated morbidity and mortality have increased dramatically as reported annual cases have more than doubled during this period [3–5].

Although much of the world faces cholera risk during pandemics, due to aquatic reservoirs [6], cholera has been endemic in Bangladesh for centuries [7] and is hyperendemic in rural Bangladesh [8]. In recent years, studies in Bangladesh have demonstrated that *V. cholerae* is the enteric pathogen most strongly linked to flood-associated diarrhea epidemics [9], and suggested that genetic susceptibility [10, 11] and socioeconomic status (SES) may be important determinants of cholera risk [10]. Studies have also reported that familial relatedness and retinol deficiency may be risk factors [11], as well as high population density [12], low education levels [12], and the proximity of household clusters to contaminated surface water [12–14]. These are in addition to well-established risk factors such as young age and poor sanitary conditions.

Most cholera risk factor studies have used case–control designs [10, 15, 16], which may have identified general diarrhea risk factors in conjunction with cholera-specific risk factors. A case-case study design could complement these prior studies by identifying cholera-specific risk factors. Case-case study designs are able overcome two perennial challenges to case–control studies: selection bias and recall bias. Compared to case–control studies, selection bias may be reduced because both the referent cases and the cases of interest are selected in the same way and represent the same population [17]. For example, in surveillance data, both sets of cases tend to be representative of those with more severe illness [18, 19] and are likely to have the same disposition to seek medical care [19]. Case-case studies are also less susceptible to recall bias than case–control studies because both the referent group and the cases of interest would have their recall stimulated by similar events [18, 20]. Since the referent group would share some diarrhea risk factors, this design is not well suited for identifying general risk factors [17], but may enable the identification of cholera-specific risk factors. Finally, case-case studies can use regularly collected surveillance data, thereby making them faster and less expensive than case–control studies [17, 18]. However, the study design also has weaknesses that must be accounted for in the interpretation of the results. Specifically, because the referent is also diseased, risk estimates cannot be extrapolated to the general population [20]. For example, the strength of association will be underestimated if a given exposure is a risk factor for both sets of cases. Conversely, the strength of association would be inflated if a given exposure was protective among the referent cases [18]. We are prevented from obtaining unbiased assessment of exposures common to both [17].

Shigellosis offers itself as an ideal referent for a case-case study of cholera since it also has a bacterial etiology (*Shigella* spp.) and is endemic in Bangladesh [21]. The epidemiology of shigellosis and cholera are both similar (e.g., they have seasonality [22, 23] and young children are at greatest risk [24, 25]) and different (e.g., a low infectious dose of shigella [26] enables direct person-to-person transmission [27]), which may lead to the identification of disease-specific risk factors and disease control interventions.

Our objective was to identify cholera-specific risk factors distinct from shigellosis risk factors. We therefore conducted a case-case study among hospitalized diarrheal patients, comparing those with cholera to those with shigellosis.

## Methods

### Study design and setting

We conducted a hospital-based case-case study using the International Centre for Diarrhoeal Disease Research, Bangladesh (icddr,b) Diarrhoeal Diseases Surveillance System (DDSS) in Matlab and Dhaka hospitals.

We selected a case-case study design for the reasons outlined in the introduction above.

The DDSS employs icddr,b hospital staff to systematically record clinical, socioeconomic, and demographic data from diarrheal patients presenting to icddr,b hospitals prior to the patients receiving their diagnoses. All DDSS patients have their stool tested for enteric pathogens, including *V. cholerae*, *Shigella*, *Salmonella*, rotavirus, amoeba, and *Giardia* species. Regardless of etiology, we considered any case of diarrhea that required hospital treatment to be severe, and focused on those cases because they are of greatest clinical importance.

The Matlab hospital DDSS is part of a larger Health and Demographic Surveillance System (HDSS) created in Matlab sub-district, a rural area in east-central Bangladesh, in 1966. The HDSS employs Community Health Research Workers to record demographic, mortality, migration, and other relevant data through bimonthly visits to each household. The Matlab HDSS catchment area covers more than 200,000 residents, with all HDSS diarrheal patients treated at the icddr,b enrolled into the DDSS. Due to river and road access, and icddr,b’s well-established relationship the with the community, use of its facilities is assumed to be homogenous throughout the study area [28].

Dhaka is the largest city in Bangladesh, with large numbers of residents living in substandard housing (slums). Since 1996, two percent of patients at the Dhaka Hospital have been systematically enrolled in the DDSS [9]. Due to the hospital’s location within the city limits, its services are considered to be accessible to all city residents. The administrative and clinical staff in Matlab and Dhaka received equivalent training to ensure comparability of care and DDSS data quality. In both settings, icddr,b hospitals provide free, high-quality diarrhea treatment.

In this case-case analysis, rural dwellers were defined as Matlab patients who were registered with the Matlab HDSS and self-reported currently living in a village. Urban dwellers were defined as Dhaka patients who self-reported currently living in slums or high-density mixed-use and residential areas.

### Study population

We analyzed in-patient and out-patient data from patients who entered icddr,b hospitals for diarrhea treatment between January 1, 2000 and December 31, 2008. Since risk factors and necessary disease control measures among children under five may be different from older individuals, their risk factors were analyzed separately [29]. We also excluded those with missing age data, non-rural Matlab patients, non-urban Dhaka patients, those with neither cholera nor shigellosis, and those with enteric co-infections. Use of anonymized data prevented us from assessing if there were multiple admissions of the same patient.

### Cholera and Shigellosis definitions

*V. cholerae* positivity was defined by the detection of *V. cholerae* O139 (Bengal), *V. cholerae* O1 El Tor Ogawa, *V. cholerae* O1 El Tor Inaba, *V. cholerae* O1 Classical Ogawa, or *V. cholerae* O1 Classical Inaba. *Shigella spp*. infection was defined by the detection of *S. dysenteriae, S. flexneri, S. boydii,* or *S. sonnei*. There were no changes in laboratory testing methods for *V. cholerae* or *Shigella spp*. during the study period.

### Data analysis

The prevalence of potential correlates of diarrhea among hospitalized patients with cholera and shigellosis at icddr,b hospitals were compared. Self-reported sociodemographic characteristics included age, sex, number of household members, education, household income, urban residence, residence in a slum community, homeownership, and presence of concrete floors in the home. Education was defined as the patient’s education (for those ≥15 years old) or the mother’s education (for those <15 years old). Self-reported water and sanitation characteristics included the patient’s household having improved toilet facilities [30], distance from the kitchen to drinking water (reported in feet and converted to meters for analysis), source of water, and drinking water treatment. Source of water was constructed by combining drinking and bathing water variables; if these were different, the least safe source was used for the analysis. Surface water was defined as that from a pond, river, or ditch. “Other” water treatment included use of tablets, filters, and sieves. Data regarding the source of water used for food preparation was unavailable for this analysis. Other potential correlates included the distance to the hospital (self-reported in miles and converted to km for analysis), the presence of a family member with diarrhea in the past week, and the season.

Clinical characteristics included general physical condition and clinical dehydration on admission as assessed by medical staff, self-reported days with diarrhea prior to admission, and the number of stools and history of vomiting in the 24 hours prior to admission. Data regarding patient deaths, if any, were not available.

### Risk factor analysis

Assessed sociodemographic risk factors included age, sex, the number of household members, years of education, monthly household income (converted from Taka using the rate of exchange at the study period’s midpoint [31]), residence in a slum community, homeownership, and the presence of concrete floors in the home. Risk factors related to sanitation and water included improved toilet facilities, distance from the kitchen to the drinking water source (10-m increments), water source, and drinking water treatment. The distance from the home to the hospital (km) and the presence of a family member with diarrhea in the past seven days were also assessed.

### Statistical methods

We used Poisson regression with robust variance estimates to calculate risk ratios (RR) and 95% confidence intervals (95% CI) for cholera hospitalization risk factors [32]. The dependent variable in the regression model was cholera hospitalization (vs. shigellosis hospitalization) and the independent variables are listed under “Risk factor analysis” above. Due to substantial differences between Dhaka and Matlab, all regression analyses were stratified by urban or rural status. Only potential risk factors with less than 5% missing data were evaluated. Stata/IC 13.1 (StataCorp LP, College Station, TX) was used for all analyses. All *P*-values are two-sided.

(Statistically significant univariable predictors (*p* < 0.10) were considered candidates for the multivariable model. Predictors with a RR between 0.9 and 1.1 were excluded from consideration for the multivariable model due to small effect sizes. Strata with less than ten observations were also excluded from consideration for the multivariable model. We used variance inflation factors (VIF) to assess collinearity among the multivariable candidates. In the event of collinearity (VIF ≥10), we considered only the more biologically plausible predictor.

We built a multivariable regression model by sequentially adding and testing statistically significant candidates from the univariable analysis, in order of effect size. Continuous variables were retained in the model if the Wald test was significant (*p* < 0.05). A categorical variable was retained if the composite linear Wald test of all the variable’s strata and the Wald test for at least one individual stratum indicator variable was significant (*p* < 0.05).

Cholera seasonality [1] and age were included in the multivariable model as a priori adjustment variables. The seasonality adjustment variable was comprised of a restricted cubic spline of the day of the year on the date of visit (1–366). The spline had seven knots and was created to prevent the imposition of artificial categories or parameters on the data [33, 34].

### Ethics statement

The Research Review Committee (RRC) and Ethical Review Committee (ERC) of the icddr,b approved the hospital surveillance activities. Due to the high proportion of illiterate patients, the icddr,b RRC and ERC waived the need for written informed consent and approved the use of oral informed consent for all participants. Parents, guardians, next of kin, or caretakers provided oral informed consent for minors. icddr,b staff documented consent in the surveillance database. All data analyses were performed using anonymized patient medical records. The University of Washington Human Subjects Division/Institutional Review Board determined this research to be exempt from human subjects review because it did not fall under the definition of human subjects research under 45CFR46.

## Results

### Study population

We excluded those with missing age (n = 45), indeterminate rural or urban residential status (n = 7,242), and age less than five years (n = 14,515). Of the remaining 11,369 patients, we excluded the 54% (n = 6,096) who had no pathogen detected, 3% (n = 365) who had laboratory-confirmed coinfection of *V. cholerae* or *Shigella spp*. with other known pathogens, and 11% (n = 1,198) who were infected with neither *V. cholerae* nor *Shigella spp*. Our final population was thus the 27% (n = 3,072) who were hospitalized with cholera and the 6% (n = 638) who were hospitalized with shigellosis. The refusal rate for participation in the DDSS was not available.

### Characteristics of hospitalized cholera and shigellosis cases

Compared to hospitalized shigellosis patients, hospitalized cholera patients were younger (median age, 24 vs. 35), more likely to be uneducated (78% vs. 70%), and less likely to be in the highest income bracket (39% vs. 51%) (Table 1). Cholera patients were also more likely to live in an urban area (66% vs. 30%), less likely to be homeowners (40% vs. 73%), and less likely to have cement floors in their homes (47% vs. 67%). With regard to sanitation and water, cholera patients were more likely to have improved toilet facilities (46% vs. 30%), to have a drinking water source within 10 meters of the kitchen (69% vs. 61%), to use tap water (58% vs. 28%), and to treat their drinking water (25% vs. 17%). In addition, cholera patients lived further from icddr,b hospitals and were more likely to have had a family member with diarrhea in the past week.

**Table 1:** **Sociodemographic, water and sanitation, and other potential correlates of hospitalization for diarrhea among icddr,b patients ≥ 5 years old, Bangladesh, 2000-2008**

	Cholera (n = 3,072)		Shigella (n = 638)	
	N	%^a^	N	%^a^
**Sociodemographic**				
**Age (yrs.)**				
5-15	772	25	96	15
16-25	999	33	143	22
26-35	530	17	89	14
36-45	326	11	84	13
46+	445	14	226	35
**Female sex**	1479	48	335	53
**No. household members**				
<3	554	18	124	19
4	580	19	111	17
5	659	21	140	22
6	484	16	98	15
7+	792	26	165	26
**Education** ^b^				
None	2391	78	447	70
1-5 years	391	13	109	17
6-9 years	219	7	52	8
>9 years	71	2	30	5
**Household income (USD/mo.)**				
>84	1205	39	324	51
50-84	1274	42	216	34
34-49	400	13	70	11
≤34	193	6	28	4
**Urban residence**	2014	666	189	30
**Residence in a slum**	342	17	25	13
**Homeownership**	1219	40	463	73
**Cement floor in home**	1446	47	426	67
**Sanitation & water**				
**Improved toilet facilities**	1414	46	188	30
**Distance to drinking water (meters)**				
0	477	16	72	11
<10	1619	53	319	50
10-19	403	13	90	14
20-50	324	11	82	13
>50	249	8	75	12
**Source of water**				
Tap	1787	58	175	28
Tube well	318	10	66	10
Surface^c^	965	31	396	62
**Drinking water treatment**				
None	2312	75	530	83
Boiling	694	23	91	14
Other	66	2	17	3
**Other potential correlates**				
**Distance to hospital (km)**				
≤3	281	9	143	22
>3 & ≤5	475	15	169	26
>5 & ≤7	422	14	86	14
>7	1892	62	240	38
**Family member with diarrhea in past week**	478	16	42	7
**Season**				
**Hot: March – June**	1177	38	180	28
**Rain: June – October**	1377	45	255	40
**Dry/Cool: October – March**	518	17	203	32

^a^Percentages may not sum to 100% due to rounding.

^b^Education of patient for those ≥15 years of age; education of mother for those <15 years old.

^c^Water from rivers, ponds, and ditches was categorized as surface water.

Regarding clinical characteristics, hospitalized cholera patients were more likely to present at icddr,b hospitals in a worse than normal general condition (85% vs. 36%) and to have clinical dehydration (94% vs. 47%) (Table 2). They were also more likely to present to a hospital within one day of diarrhea onset (72% vs. 45%) and to have stool without blood or mucus (96% vs. 40%). In the 24 hours prior to admission, they were somewhat more likely to have 15 or fewer stools (79% vs. 75%) and much more likely to have vomited (93% vs. 53%).

**Table 2:** **Clinical characteristics of patients ≥ 5 years old with hospitalized diarrhea in icddr,b hospitals, Bangladesh, 2000–2008**

	Cholera (n = 3,072)		Shigella (n = 638)	
	N	%^a^	N	%^a^
**General condition**				
Normal	463	15	406	64
Restless	274	9	73	11
Lethargic but irritable when touched	857	28	140	22
Drowsy/cold & sweating extremities	1475	48	19	3
**Clinical dehydration**				
None	198	6	338	53
Some	1003	33	275	43
Severe	1868	61	25	4
<1	2217	72	289	45
1-6	843	28	338	53
7-14	11	0	7	1
15+	0	0	4	1
**Stool contents**				
Usual	2934	96	256	40
Mucus	110	4	129	20
Blood	3	0	31	5
Mucus and blood	25	1	222	35
**# Stools in 24 hours**				
3-5	192	6	42	7
6-10	1283	42	276	43
11-15	967	31	162	25
16-20	353	12	70	11
21+	277	9	88	14
**Vomiting in last 24 hours**				
None	214	7	298	47
<10 times	2347	76	324	51
10+ times	511	17	16	3

^a^Percentages may not sum to 100% due to rounding.

### Risk factor analysis

Univariable risk estimates for icddr,b diarrheal patients, stratified by rural or urban residence, are reported in Table 3. All variables had less than 5% missing data and there was no evidence of collinearity among the variables. Education, household income, homeownership, having cement floors in the home, sources of water, and having a family member with diarrhea in the past week were statistically significant and had a RR less than 0.90 or greater than 1.10 in the rural setting. In the urban setting, only education was significant and had a RR less than 0.90 or greater than 1.10.

**Table 3:** **Univariable risk factor analysis for cholera compared to shigellosis among patients ≥ 5 years old hospitalized for diarrhea, icddr,b hospitals, Bangladesh, 2000–2008**

	Rural univariable			Urban univariable		
	(n = 1,507)			(n = 2,203)		
	Cholera/Total (%)	RR	95% CI	Cholera/Total (%)	RR	95% CI
**Sociodemographic**						
**Age (10 yr. intervals)**	-	0.94	(0.92-0.95)	-	0.97	(0.96-0.98)
**Female sex**	588/845 (70)	0.98	(0.92-1.05)	891/969 (92)	1.01	(0.98-1.04)
**No. household members**	-	1.03	(1.01-1.04)	-	1	(1.00-1.00)
**Education** ^a,b^						
None	782/1095 (71)	1	-	1609/1743 (92)	1	-
1-5 years	181/268 (68)	0.95	(0.86-1.04)	210/232 (91)	0.98	(0.94-1.02)
6-9 years	84/120 (70)	0.98	(0.87-1.11)	135/151 (89)	0.97	(0.92-1.02)
>9 years	11/24 (46)	0.64	(0.41-0.99)	60/77 (78)	0.84	(0.75-0.95)
**Household income (USD/mo.)** ^c^						
>84	416/647 (64)	1	-	789/882 (89)	1	-
50-84	494/657 (75)	1.17	(1.09-1.26)	780/833 (94)	1.05	(1.02-1.08)
34-49	127/168 (76)	1.18	(1.06-1.30)	273/302 (90)	1.01	(0.97-1.06)
≤34	21/35 (60)	0.93	(0.71-1.23)	172/186 (92)	1.03	(0.99-1.08)
**Residence in a slum**	-	-		342/367 (93)	1.02	(0.99-1.06)
**Homeownership**	1045/1480 (71)	1.41	(0.96-2.08)	174/202 (86)	0.94	(0.88-0.99)
**Cement floor in home**	70/122 (57)	0.80	(0.69-0.94)	1555/1714 (91)	0.97	(0.94-0.99)
**Sanitation & Water**						
**Improved toilet facilities**	78/122 (64)	0.90	(0.79-1.04)	1336/1480 (90)	0.96	(0.94-0.99)
**Dist. to drinking water (10 m increments)**	-	1.01	(1.00-1.01)	-	1	(1.00-1.01)
**Source of water**						
Tap	3/3 (100)	1.50	(1.34-1.67)	1784/1959 (91)	0.96	(0.93-1.00)
Tube well	108/162 (67)	1	-	210/222 (95)	1	-
Surface^d^	947/1342 (71)	1.06	(0.94-1.19)	18/19 (95)	1.00	(0.90-1.12)
**Drinking water treatment**						
None	1000/1426 (70)	1	-	1312/1416 (93)	1	-
Boiling	8/15 (53)	0.76	(0.47-1.22)	686/770 (89)	0.96	(0.93-0.99)
Other	50/66 (76)	1.08	(0.94-1.24)	16/17 (94)	1.02	(0.90-1.15)
**Other potential correlates**						
**Distance to hospital (km)**	-	1.02	(1.01-1.02)	-	1	(1.00-1.00)
**Family member with diarrhea in past week**	162/194 (84)	1.22	(1.14-1.32)	316/326 (97)	1.07	(1.05-1.10)

^a^Education of patient for those ≥15 years of age; education of mother for those <15 years old.

^b^Statistical evidence of a decreasing trend among urban residents (*P* = 0.002).

^c^Statistical evidence of an increasing trend among rural residents (*P* = 0.003).

^d^Water from rivers, ponds, and ditches was categorized as surface water.

In the rural multivariable model, risk for cholera hospitalization decreased with increasing levels of education, with those having more than nine years of education experiencing approximately 40% reduced risk (adjusted Risk Ratio [aRR] = 0.61, 95% confidence interval [CI]: 0.40-0.93) compared to those with no education (Table 4). Those who had a family member with diarrhea in the past week had a 17% increase in risk for cholera hospitalization (aRR = 1.17, 95% CI: 1.09-1.26).

**Table 4:** **Multivariable risk factor analysis for cholera compared to shigellosis among patients ≥ 5 years old hospitalized for diarrhea, icddr,b hospitals, Bangladesh, 2000–2008**

	Rural multivariable^a^		Urban multivariable^a^	
	(n = 1,507)		(n = 2,203)	
	RR	95% CI	RR	95% CI
**Sociodemographic**				
**Education** ^b^				
None	1		1	
1-5 years	0.85	(0.78-0.92)	0.97	(0.93-1.02)
6-9 years	0.86	(0.76-0.97)	0.96	(0.91-1.01)
>9 years	0.61	(0.40-0.93)	0.84	(0.75-0.94)
**Other potential correlates**				
**Family member with diarrhea in past week**	1.17	(1.09-1.26)	-	

^a^Adjusted for a priori confounders (age and season) and other predictors in the model.

^b^Education of patient for those ≥15 years of age; education of mother for those <15 years old. There is statistical evidence of decreasing risk with increasing levels of education in both settings (*P* < 0.001).

In the urban multivariable model, education also was associated with cholera hospitalization risk (*P* = 0.07), with those having more than nine years of education experiencing 16% reduced risk (aRR = 0.84, 95% CI: 0.75-0.94), compared to those without an education (Table 4). Based upon our predetermined criteria, no other variables were included in the model except for age and season, the a priori adjustment variables.

We performed cross-tabulations of sources of water and improved toilet facilities, stratified by urban and rural residence and education level (Table 5). We did this in order to explore the relationship between water source and education variables, both of which were expected to be correlated with cholera hospitalization. In both settings, higher levels of education were associated with improved toilet facilities. The majority of those with improved toilets in Matlab continued to use surface water, but the majority of those with improved toilets in Dhaka used tap water.

**Table 5:** **Cross tabulations of toilet facilities and sources of water, stratified by urban and rural residence and education level**

		Rural						Urban					
		Improved		Unimproved				Improved		Unimproved			
		Toilet		Toilet				Toilet		Toilet			
Education	Water source	n	%^a^	n	%^a^	Total	*P* ^b^	n	%^a^	n	%^a^	Total	*P* ^b^
	**Tap**	3	0.2	0	-			1,391	63.2	568	25.8		
**All levels**	**Tube well**	43	2.9	119	7.9			81	3.7	141	6.4		
	**Surface**	76	5.0	1,266	84.0	1,507	<0.001	5	0.2	14	0.6	2,200	<0.001
	**Tap**	1	0.1	0	-			1,007	57.8	525	30.2		
**None**	**Tube well**	12	1.1	71	6.5			66	3.8	126	7.2		
	**Surface**	49	4.5	962	87.9	1,095	<0.001	4	0.2	13	0.7	1,741	<0.001
	**Tap**	1	0.4	0	-			181	78.0	27	11.6		
**1-5 years**	**Tube well**	13	4.9	31	11.6			11	4.7	11	4.7		
	**Surface**	15	5.6	208	77.6	268	<0.001	1	0.4	1	0.4	232	<0.001
	**Tap**	0	-	0	-			132	87.4	13	8.6		
**6-9 years**	**Tube well**	13	10.8	13	10.8			3	2.0	3	2.0		
	**Surface**	10	8.3	84	70.0	120	<0.001	0	-	0	-	151	0.016
	**Tap**	1	4.2	0	-			71	93.4	3	3.9		
**>9 years**	**Tube well**	5	20.8	4	16.7			1	1.3	1	1.3		
	**Surface**	2	8.3	12	50.0	24	0.037	0	-	0	-	76	0.103

^a^Percentage of the total.

^b^ *P*-value from Fisher’s exact test.

## Discussion

Higher levels of education were correlated with reduced risk for cholera hospitalization in both rural and urban Bangladesh. In addition, having a family member with diarrhea in the past week was associated with increased risk among rural dwellers.

The association between increasing education levels and decreasing cholera hospitalization risk was expected based on prior studies in Matlab [12, 29]. There is evidence from other contexts that shigellosis may also be associated with low levels of education [35]. Therefore, given our case-case study design, the magnitude of the risk reduction attributable to higher education levels may be attenuated compared to what would be seen with population-based controls. The exact mechanism(s) through which education affects cholera risk have not been determined and are worthy of further study. However, the results of the exploratory cross tabulations of toilet facilities and water sources by education level suggest that part of the protective effect of higher education may be mediated through these variables.

The increased risk associated with family members having had diarrhea in the past week was expected, and is likely due to household-level exposures to *V. cholerae*-contaminated water and food [36]. In addition, family members have similar genetics and may share an elevated susceptibility to cholera infection [10, 11, 37] as well as severe cholera disease. However, unlike *Shigella* [27], which is easily passed from person-to-person, the contribution of person-to-person cholera transmission among family members remains unclear [2, 38].

Improved toilet facilities are generally considered to be strongly protective for cholera. However, we observed no substantial protective effect for cholera hospitalization as opposed to shigellosis hospitalization in either the rural (where few had improved toilet facilities) or the urban setting. This is most likely due to attenuation of the risk estimate because improved toilet facilities reduce the risk for multiple diarrhea etiologies. As mentioned above, the inability to identify general risk factors is a limitation of the case-case study design. However, it is also possible that homes with improved toilet facilities continued to have high levels of fecal contamination [39], thereby negating some of the potential benefit of the infrastructure.

Based on our previous study among children under five in which we compared those hospitalized with cholera to those hospitalized with all other diarrhea [29], we anticipated that low SES would be associated with increased risk for cholera hospitalization. However, household income, homeownership, and other measures of SES (except for education) were not significant in our multivariable models. This might be because SES is only related to cholera risk among children under five, or because SES impacts risk for cholera and shigellosis equally.

Given the substantial differences in the availability of improved toilets, sources of water, family income, home ownership, and numerous unmeasured geographic and social differences between Matlab and Dhaka, it is surprising that we found little evidence for differential transmission patterns. However, the increased risk for cholera hospitalization associated with having a family member with diarrhea in the past week was only a risk factor in the rural setting. One possible explanation for why this association is weaker in Dhaka may be that urban dwellers are more likely to eat and drink outside of their home, whereas Matlab residents are more likely to share the contaminated sources of food or drink. Unfortunately, we are unable to assess this with our data.

### Strengths and limitations

The strengths of our study include reliable *V. cholerae* and *Shigella spp.* diagnosis by experienced clinical laboratories, methodical sampling, and a large sample size. The case-case study design is also a strength in that it isolates diarrhea risk factors unique to cholera hospitalization [17]. However, this study design is also limited in that cholera shares some risk factors with shigellosis, so our risk estimates are likely to be attenuated compared to those we would have obtained if we had used a population without diarrhea as a comparison group. Another limitation was the use of anonymized data, which prohibited us from identifying repeat patient visits and patients from the same family or household [11]. Both conditions would violate independence assumptions underlying our statistical analyses. However, based on the natural immunity and low re-hospitalization rates reported in prior studies [7, 40], few repeat cholera visits would be expected. In addition, any family or household clustering effects are expected to be relatively small in comparison to the study sample size. The large number of patients with no pathogen detected is another limitation. Some of the patients excluded for having no pathogen detected may actually have had *V. cholerae* or *Shigella* infection, and excluding them may have biased our sample. Performing polymerase chain reaction (PCR) testing on a subsample of these patients’ specimens might have given an indication of what proportion of cholera and shigellosis cases were misclassified on this account. However, this assumes that PCR is more sensitive than culture --- whereas some evidence suggests it is not [41].

## Conclusions

We report that lack of education is a risk factor specific for cholera hospitalization in both rural and urban Bangladesh. In addition, having a family member with diarrhea in the past seven days is a risk factor in rural Bangladesh. Further studies are needed to elucidate the pathway through which education impacts cholera risk in order to create targeted interventions in cholera-endemic areas. In addition, among rural families with an index case of diarrhea, interventions to facilitate hygienic practices should be assessed as a means to reduce the incidence of secondary cases at the household level.

## Abbreviations

aRR: Adjusted risk ratio
CI: Confidence intervals
DDSS: Diarrhoeal diseases surveillance system
ERC: Ethical Review Committee
HDSS: Health and demographic surveillance system
Icddr: b: International Centre for Diarrhoeal Disease Research, Bangladesh
ORS: Oral rehydration solution
PCR: Polymerase chain reaction
RR: Risk ratios
RRC: Research Review Committee
SES: Socioeconomic status
VIF: Variance inflation factors
